# Protective Effects of Selected Botanical Agents on Bone

**DOI:** 10.3390/ijerph15050963

**Published:** 2018-05-11

**Authors:** James Jam Jolly, Kok-Yong Chin, Ekram Alias, Kien Hui Chua, Ima Nirwana Soelaiman

**Affiliations:** 1 Department of Pharmacology, Faculty of Medicine, Pusat Perubatan Universiti Kebangsaan Malaysia, Jalan Yaacob Latif, Bandar Tun Razak, Cheras 56000, Wilayah Persekutuan Kuala Lumpur, Malaysia; jamesjamjolly@yahoo.com.my (J.J.J.); chinkokyong@ppukm.ukm.edu.my (K.-Y.C.); 2 Department of Biochemistry, Faculty of Medicine, Pusat Perubatan Universiti Kebangsaan Malaysia, Jalan Yaacob Latif, Bandar Tun Razak, Cheras 56000, Wilayah Persekutuan Kuala Lumpur, Malaysia; ekram.alias@ppukm.ukm.edu.my; 3 Department of Physiology, Faculty of Medicine, Pusat Perubatan Universiti Kebangsaan Malaysia, Jalan Yaacob Latif, Bandar Tun Razak, Cheras 56000, Wilayah Persekutuan Kuala Lumpur, Malaysia; ckienhui@gmail.com

**Keywords:** bone remodelling, complementary therapies, herbal medicine, osteoblast, osteoclast

## Abstract

Osteoporosis is a serious health problem affecting more than 200 million elderly people worldwide. The early symptoms of this disease are hardly detectable. It causes progressive bone loss, which ultimately renders the patients susceptible to fractures. Osteoporosis must be prevented because the associated fragility fractures result in high morbidity, mortality, and healthcare costs. Many plants used in herbal medicine contain bioactive compounds possessing skeletal protective effects. This paper explores the anti-osteoporotic properties of selected herbal plants, including their actions on osteoblasts (bone forming cells), osteoclasts (bone resorbing cells), and bone remodelling. Some of the herbal plant families included in this review are Berberidaceae, Fabaceae, Arecaceae, Labiatae, Simaroubaceaea, and Myrsinaceae. Their active constituents, mechanisms of action, and pharmaceutical applications were discussed. The literature shows that very few herbal plants have undergone human clinical trials to evaluate their pharmacological effects on bone to date. Therefore, more intensive research should be performed on these plants to validate their anti-osteoporotic properties so that they can complement the currently available conventional drugs in the battle against osteoporosis.

## 1. Introduction

Osteoporosis is a metabolic bone disorder resulting from an imbalance of bone remodelling, in which the rate of bone resorption is higher than the rate of bone formation [1,2]. In turn, this gives rise to low bone mass, microarchitectural deterioration, and eventually an increased risk for fragility fractures [1,2,3]. Osteoporosis can be classified into primary (Type I and II) and secondary osteoporosis. Primary type I osteoporosis occurs in women soon after menopause (postmenopausal osteoporosis) and in men during and after middle-age [4]. On the other hand, primary type II or senile osteoporosis is due to old age. Both sexes may develop primary type II osteoporosis over the age of 70, whereby both trabecular and cortical bones degenerate, thus causing proximal femora, vertebrae, and radii fractures. Women have a two-fold higher risk than men to suffer from primary type II osteoporosis due to their low peak bone mass [2,4,5,6]. Secondary osteoporosis is due to medications or certain medical conditions, such as hypogonadism, hyperparathyroidism, or leukemia [7]. Prolonged use of some medications can lead to bone loss, such as oral or high-dose inhaled corticosteroids, thyroid hormone replacement, and aromatase inhibitors [7,8,9]. Osteoporosis is closely associated with increased mortality due to complications of osteoporotic fractures, particularly at the vertebrae and hips [2,10,11].

Most current therapies for osteoporosis focus on inhibiting bone resorption and reducing bone remodelling [12,13]. Parathyroid hormone, and its analogue teriparatide, are the only anabolic therapies available to treat severe osteoporosis [14]. The current drug therapies have been proven to improve bone mineral density and reduce fracture risk, but prolonged use has been associated with various side effects [15,16]. Therefore, the search for new drugs is ongoing [17,18]. In addition, the prophylactic agents for osteoporosis are limited to calcium and vitamin D. Recent advancement in phytomedicine has stimulated interests to transform herbal plants into treatment for chronic diseases, like osteoporosis [2,12,19]. Some vigorously studied herbal plants have demonstrated antiosteoporotic effects in cellular and animal studies [13,19,20]. These include *Rhizoma alismatis* [21], *Curculiginis rhizoma* [22], *Hemidesmus indicus* (L). R. Br [23], *Passiflora foetida* [24], *Cissus quadrangularis* [25], and *Dalbergia sissoo* [26].

In this paper, selected herbal plants which have demonstrated skeletal protecting effects in scientific studies were reviewed. Their geographical origin, active chemical components, and mechanism of action were discussed. The herbal plants included in this review were tested at least in animal or cellular (cultured osteoblasts and osteoclasts) studies, and their bioactive constituents had been identified. Six plant families originating from the Asian continent were discussed, namely Berberidaceae (East Asia), Fabaceae (East Asia), Arecaceae (Southeast Asia), Labiatae (Southeast Asia), Simaroubaceaea (Southeast Asia), and Myrsinaceae (Southeast Asia).

## 2. Antiosteoporotic Constituents Extracted from Natural Plants

### 2.1. The Berberidaceae Family

Epimedium plants (a genus of flowering plants from the Berberidaceae family) are low-growing and deciduous perennial plants [27,28,29]. They are also known as barrenwort, fairy wings, and bishop’s hat. The leaves of other species such as *Epimedium brevicornum* Maxim, *Epimedium sagittatum* Maxim, *Epimedium pubescens* Maxim, and *Epimedium koreanum* Nakai have been used traditionally to combat osteoporosis and menopause-related diseases in China [27,30,31,32]. These herbal medicinal plants are used throughout the ages as an antiosteoporotic agent in Chinese traditional medicine [27,30,31,32]. The crude extract of Epimedium flavonoids contain icariin, epimedin B, and epimedin C. These compounds have been identified as the main antiosteoporotic constituents of Epimedium plants by inhibiting bone resorption, triggering bone formation, and blocking urinary calcium excretion [27,30,31,32]. They have also been shown to prevent osteoporosis without causing uterine hyperplasia in the ovariectomized rat model [20,27,30,31].

The Epimedium flavonoids possess estrogenic activity and improve the maturation of osteoblasts by inducing the expression of alkaline phosphatase (ALP), bone morphogenetic protein-2 (BMP-2) and core binding factor α1 (Cbfα1). They also increase expression of osteoprotegerin (OPG) but reduce the expression of receptor activator of nuclear factor-κB ligand (RANKL), thereby inhibiting the formation of osteoclasts [27,30,31,32,33]. Several studies also showed that Epimedium flavonoids upregulated expressions of BMP or Wingless-type signalling (Wnt-signaling) pathway related regulators, like cyclin D [20,27,30,31].

Icariin has been identified as the most active flavonoid glucoside extract of Epimedium plant [27,31]. Icariin inhibits bone loss in the distal femur and tibia in ovariectomized rat models [20,27,30,31]. It is suggested that icariin activates estrogen receptor (ER) and induces ER-dependent bone activity [20,27,30,31]. Icariin also decreases the tartrate-resistant acid phosphate activity (TRAP) activity of osteoclasts, their size and bone resorption activity. This is achieved by lowering IL-6 and TNF-α expression [20,27,30,31]. Icariin can inhibit cyclooxygenase type-2 (COX-2) activity, expression of LPS-induced hypoxia inducible factor-1α (HIF-1α), and activation of the p38 and c-Jun N-terminal kinase (JNK) in osteoclasts [20,27,30,31]. It also inhibits osteoclasts differentiation by reducing ERK1/2 and I*κ*-Bα LPS-induced activation [20,27,30,31].

Ikarisoside A is a natural flavonoid extracted from Epimedium species of *E. koreanum*. It possesses antioxidant and anti-inflammatory properties in LPS-stimulated bone marrow-derived macrophage precursor cells and in RAW264.7 cells [20,30,31]. It also inhibits the formation of osteoclasts and bone resorption activity from these precursor cells [20,31]. Moreover, Ikarisoside A reduces the expression of osteoclastic genes, such as TRAP, matrix metalloproteinase 9 (MMP-9), cathepsin K, and receptor activator of NF-κB (RANK) [20,30,31]. This is achieved by suppressing the activation of the nuclear factor kappa-light-chain-enhancer of activated B cells (NF-κB), JNK, and protein kinase B (Akt)-RANKL [20,30,31]. Thus, it can be concluded that Ikarisoside A has the potential to be used as a remedy to treat diseases involving rheumatoid arthritis and osteoporosis [20,30,34]

### 2.2. The Fabaceae Family

The soybean, scientifically known as *Glycine max* L. (Fabaceae), is mainly grown in Southwest Asia [27]. It is a rich source of proteins and flavonoids, such as daidzein, biochanin A, and genistein [27]. Supplementing soybean protein in the diet is effective in reducing the loss of bone mineral density in ovariectomized rats [27,35,36]. In animal models of bone loss, isoflavones can preserve trabecular microstructure [27,37]. They act by modulating gene expression of collagen type I (COL I), osteocalcin, calciotropic receptor, ALP, cytokines, and growth factors [27,38]. The phytoestrogens in soybean have been shown to exert significant effects on bone metabolism in postmenopausal women. It could be used as a dietary supplement to prevent postmenopausal osteoporosis since isoflavones can improve bone turnover markers, bone mineral density, and bone strength among postmenopausal women [27,36,37,38]. However, the skeletal effects of soy isoflavones supplementation in humans remain debatable because several meta-analyses reported that the effects were minimal [39,40]. Nevertheless, further studies are necessary to verify the magnitude of the skeletal effects of soy isoflavones in humans.

Genistein is an isoflavone exhibiting estrogenic effect on bone. It modulates B-lymphopoiesis in bone marrow and inhibits bone degradation without any estrogenic effect in the uterus [27,41]. The antiosteoporotic effects of flavonoids depend on the mixture of their estrogenic agonist–antagonist properties [27,41]. Other studies suggest that the antiosteoporotic effects may be derived from other biochemical properties of flavonoids, including enzymatic inhibition of certain protein kinases or activation of estrogen type I receptors [27]. The clinical effectiveness of the flavonoids may be dependent on their ability to produce equol, an isoflavandiol metabolized by gut microflora from daidzein [27,42]. It shows a higher estrogenic activity than the predominant flavonoids [27,42].

Herbal plants of the species *Psoralea corylifolia* L. (commonly known as Malay Tea, Cot Chu, or Ku Tzu locally) belongs to the family Fabaceae [27]. The fruit of this plant is used traditionally to treat bone fractures, osteomalacia, osteoporosis, and joint disorders [13,43]. The fruit extract of *P. corylifolia* significantly increases the serum concentration of inorganic phosphorus and induces bone calcification in rats [27,43]. The crude extracts of its fruit and seed, as well as two of its dominant isoflavones (corylin and bavachin), have been found to stimulate bone formation [27,43]. Extracts of *P. corylifolia* from different parts of the plants also contain bakuchalcone, psoralen, bakuchiol, psoralidin, bavachinin, isopsoralen, and flavones [44].

Some bioactive compounds isolated from *P. corylifolia* have been found to exert bone-protective effects. Bavachalcone can inhibit osteoclastogenesis by hindering the ERK and Akt signaling, as well as Chromosome-Fos (c-Fos) and nuclear factor of activated T cells c1 (NFATc1) induction during differentiation [27,43]. Psoralidin, bakuchiol, isobavachin, and corylin have been found to have strong antioxidant activities, whereas other compounds, such as bavachin and corylin, have been shown to stimulate osteoblastic proliferation [13,27]. Bakuchiol has a three-fold higher binding affinity for estrogen receptor alpha (ERα) than for estrogen receptor beta (ERβ) [13,27]. It does not have significant uterotrophic activity, although demonstrating in vitro estrogenic activity [27,45]. It can reduce postmenopausal bone loss by increasing ALP, calcium concentrations, serum estrogen concentration, and bone mineral density [27,45]. Psoralen, a coumarin-like derivative extracted from the fruit of *P. corylifolia* L., has stimulatory effects on new bone formation [27,46,47]. It also modulates differentiation of osteoblasts in a dose-dependent manner in primary mouse calvariae by upregulating osteoblast-specific genes expression of osteocalcin, type I collagen, and sialoprotein [46,47]. Psoralen affects BMP signalling activation in order to promote differentiation of osteoblasts [46,47,48]. It stimulates BMP-2 and BMP-4 gene expression, as well as increases phospho-Smad1/5/8protein level [46,47,48]. This evidence suggests that psoralen is a potent anabolic agent in treating osteoporosis [46,47,48].

### 2.3. The Arecaceae Family

Oil palm in the palm family (Arecaceae) is mostly cultivated as a source of oil [49]. Oil palm is grown extensively in the equator region of native West and Central Africa, as well as in Asian countries including Malaysia and Indonesia [50]. The most planted species of Arecaceae Family is *Elaeis guineensis* (African oil palm) and other species such as *Elaeis oleifera* (American oil palm) and *Attalea maripa* (Maripa palm) are lesser known [51]. Palm oil is an edible vegetable oil derived from the mesocarp (orange-red pulp) of the oil palm fruits [49]. It is naturally reddish in colour due to the presence of high beta-carotene content [52,53].

Palm oil of *Elaeis guineensis* is well known to have high content of vitamin E [49]. Vitamin E is a conjoint term for tocopherol and tocotrienol isoforms which are well-known for their antioxidant and anti-inflammatory properties as well as other beneficial effects on the body [54,55]. Both isoforms of tocopherols and tocotrienols exist in four different forms in nature: namely; α-, β-, γ-, and δ- [55,56]. In nature, these isomers are normally present as a mixture of varying composition [57]. For example, vitamin E extracted from crude palm oil consists of around 36% α-tocopherol, and the rest are made up by the four tocotrienol isomers [58]. On the other hand, vitamin E from annatto extract comprises of approximately 90% δ-tocotrienol and the rest is γ-tocotrienol [59].

The anti-oxidative and anti-inflammatory properties of tocotrienol make it a suitable anti-osteoporotic agent [60,61]. Both oxidative stress and inflammation are known to be involved in the pathogenesis of osteoporosis [62,63]. Oxidative stress has been shown to harm osteoblasts by affecting their differentiation and survival rate [64]. Additionally, oxidative stress also enhances the signalling of osteoclasts and simultaneously promotes their differentiation [65]. Proinflammatory cytokines—such as interleukin-1, interleukin-6, and tumour necrosis factor α—are also increased by oxidative stress, and they are also harmful to the bone [66].

A study by Hermizi et al. (2009) has shown that, both tocotrienol-rich fraction and gamma-tocotrienol supplementations were effective in retaining trabecular bone structure in nicotine-induced bone loss model [67]. Also, Aktifanus et al. (2012) and Soelaiman et al. (2012) have reported that, supplementation with tocotrienol reduced single-labelled surface and increased double-labelled surface in the ovariectomized rats [68,69]. In addition, ovariectomized rats supplemented with 30 and 60 mg/kg body weight of palm vitamin E had shown significantly higher bone mineral density at the femur and vertebrae as compared to the control untreated group [70]. Similar findings were reported in the testosterone deficiency, buserelin, and glucocorticoid-induced bone loss model [71,72,73,74,75,76]. Studies also have shown that palm vitamin E was able to restore bone calcium levels in the femur and vertebra of orchidectomized and ovariectomized rats [70,71].

The skeletal effects of vitamin E have been tested in many human studies but in most cases synthetic alpha-tocopherol was used (reviewed in [77,78]). The efficacy of palm vitamin E mixture rich in tocotrienol in preventing osteoporosis has not been studied so far. A similar vitamin E mixture, also rich in tocotrienol, from annatto beans has been tested by Shen et al. (2018) [79]. The results showed that tocotrienol decreased bone resorption markers and oxidative stress in post-menopausal osteopenic women after 12 weeks [79].

### 2.4. The Labiatae Family

A Chinese herb known as *Salvia miltiorrhiza* Bunge (commonly known as ‘dan shen’ or ‘red sage root’) from the family of Labiatae is traditionally used to treat diseases related to cardio-cerebral disorders [48,80,81]. *S. miltiorrhiza* has been shown pharmacologically to possess anticoagulation, blood flow improvement, anti-inflammatory, free radical scavenging, and mitochondrial protective properties [48,81,82]. Phytochemical studies of *S. miltiorrhiza* Bunge have revealed multiple groups of compounds, including tanshinones (tanshinone I, tanshinone IIA, 16-dihydrotanshinone I, cryptotanshinone) and phenolics (salvianolic acid A, protocatechuicaldehyde, and salvianolic acid B) [27,83,84]. Treatment with *S. miltiorrhiza* significantly prevents the decrease in trabecular bone mass and bone mineral density, reduces TRAP activity and parameters of oxidative stress, which includes malondialdehyde (MDA) and nitric oxide (NO) induced by sex hormones deficiency in rodents [20,27,82]. Tanshinones are reported to reduce the TRAP-positive multinucleated osteoclast formation [85]. Tanshinone IIA is proven to partially inhibit ovariectomy-induced bone loss by reducing bone turnover in vivo [27,85,86]. It inhibits osteoclast formation by suppressing the c-fos and NFATc1 expression induced by RANKL [27,85,86].

Salvianolic acid A from *S. miltiorrhiza* Bunge can inhibit bone loss in rats given long-term prednisone [20,87]. This is achieved by regulating osteogenesis and suppressing adipogenesis in bone marrow stromal cells [20,87]. Similarly, Salvianolic acid B has been used to inhibit glucocorticoid-induced cancellous bone loss and suppress adipogenesis [20]. It modulates the differentiation of bone marrow stromal cell (MSC) to osteoblasts and upregulates osteoblastic activities. It decreases the differentiation of glucocorticoid-associated adipogenesis through modulating the expression of Dickkopf-1, RUNX2, peroxisome proliferator-activated receptor-gamma (PPAR-γ), and β-catenin in MSC [20,88].

### 2.5. The Simaroubaceae Family

Tongkat Ali, also known as *Eurycoma longifolia*, from the family Simaroubaceae, is a traditional herbal plant found in Malaysia [89,90]. The root extract of Tongkat Ali is a well-known folk remedy among the Malaysians used to enhance fertility and sexuality, and delay ageing [89]. The bioactive compounds of these plants contain quassinoid alkaloids which are believed to cure allergies, relieve fevers, reduce tumours, and treat malaria [89,91]. Other bioactive compounds found in this plant are tannins and high-molecular-weight glycoproteins, polysaccharides and mucopolysaccharides [89].

Eurycomalactone, eurycomanone, and eurycomanol of *E. longifolia* have been shown to increase testosterone level in the blood and are capable of inhibiting the sex hormone-binding globulin [89,92,93]. Testosterone is known to enhance bone formation and prevent osteoporosis [89,94,95]. Testosterone and 5-α-dihydrotestosterone suppress RANKL and the number of colony-forming unit-macrophages, thereby reducing osteoclast numbers [96]. Consequently, the bone degradation process will be halted and bone density will be maintained [92,93]. Testosterone replacement increases bone density and mass and is an effective treatment for male osteoporosis due to hypogonadism [89,93,94,95,97]. However, it comes with some side effects, such as increased risk for prostate cancer, polycythemia, and cardiovascular events [98]. *E. longifolia*, as an androgenic compound, may act as an alternative to prevent osteoporosis associated with low testosterone level [89,92,93]. It has a good safety profile and convenient oral administration [89,92,93].

### 2.6. The Myrsinaceae Family

The herbal plant traditionally known as Kacip Fatimah (*Labisia pumila*) belongs to the family *Myrsinaceae* [99,100]. *Labisia pumila* water extract is traditionally used by Malay women to treat menstrual irregularities and dysmenorrhoea [99,100]. It is also used to improve uterine contraction post-delivery and to promote sexual function [99,100]. Its water extract is also being consumed to treat diseases such as gonorrhoea, rheumatism, dysentery, and bone disorders [101]. The plant *L. pumila* is capable of inducing the production of estrogen. Post-menopausal women are prone to have osteoporosis due to decreased circulating estrogen [100,102]. Estrogen induces osteoclast apoptosis and inhibits osteoblast apoptosis [99,100]. This reduces bone degradation and increases bone formation activity [99,100].

Pro-inflammatory cytokines, such as IL-1 and IL-6, are capable of influencing osteoclastogenesis by self-renewal stimulation [101]. These pro-inflammatory cytokines are inhibited by the presence of estrogen [99,101,102]. According to recent studies, *L. pumila* is capable of inducing the production of estrogen. Therefore, *L. pumila* can be regarded as an alternative to estrogen replacement therapy (ERT) [99,101,102].

Also, *L. pumila* exerts anti-oxidant properties due to the presence of active compounds, such as ascorbic acid, anthocyanin, beta-carotene, flavonoids, and phenolic compounds [101,102]. Other active constituents of *L. pumila*, such as anthocyanin and phenolics, also play a role as anti-oxidant and anti-inflammatory agents [101,102]. These effective free radical scavengers can help to improve chronic diseases related to oxidative stress [102].

## 3. Perspectives

Several important issues should be considered when using natural herbal plants to treat osteoporosis. These issues are (i) selectivity: the mechanism of action, selective binding to sites of action and any possible resistance of the compound towards bioactive site action; (ii) therapeutic/pharmaceutical index: the benefit-to-risk ratio of the applied bioactive compound and clinical trials before being used as a standard therapy or along with standard therapy; (iii) controllability: the rate of targeted bioactive compound must be clear, reproducible and controllable; and lastly, (iv) convenience: preferably, the drug should be orally administered; therefore the liquid or tablet dosage form must be initially formulated and stabilized, making it easier to be taken orally [22].

The safety of herbal remedies should also be studied intensively. There is a widespread belief that herbals are natural and harmless. However, studies have shown that hepatotoxicity is the most frequently reported toxic effect of herbal remedies [22,100]. Therefore, precise investigation of the bioactive compounds and scientific data regarding the safety and toxicity are needed before definite clinical trials are conducted.

In addition, standardization of medical herbal plants should also be emphasized. The lack of standardization has contributed to difficulties in validating the efficacy of the plants, which is important for further study of targeted bioactive compounds. Plants that are commonly used in laboratory experiments should be investigated thoroughly in terms of their pharmacology and therapeutic effect before being tested in patients suffering from osteoporosis and other bone-related diseases.

Many natural herbal plants have the potential to be developed as anti-osteoporotic agents. However, only a fraction of these plants has been thoroughly investigated by researchers. More reliable, efficient, and rapid bioassays should be developed to examine the antiosteoporotic efficacy of these botanical extracts, as well as to identify the compounds responsible for the bone-protective effects and mechanism involved. Most anti-osteoporotic agents derived from herbal medicinal plants can be used as prophylactic rather than therapeutic agents. If no clinical trials are done, the application and development of these herbal plants will remain restricted and undiscovered. It is important to translate laboratory findings to clinical outcomes to enable drugs from natural plants to be used for human therapy.

There are some limitations pertaining to the discussion of this review. Quality assessment was not performed on the studies included in this review. Therefore, some studies quoted might be subjected to biases and errors. The readers should interpret the studies with caution. Most botanical agents cited do not have a complete safety profile, either in animal or in humans. In most animal studies, the efficacy data of these botanical agents are not complemented with safety data. Therefore, the therapeutic index of these agents remains elusive to the readers.

## 4. Conclusions

**Figure 1:**
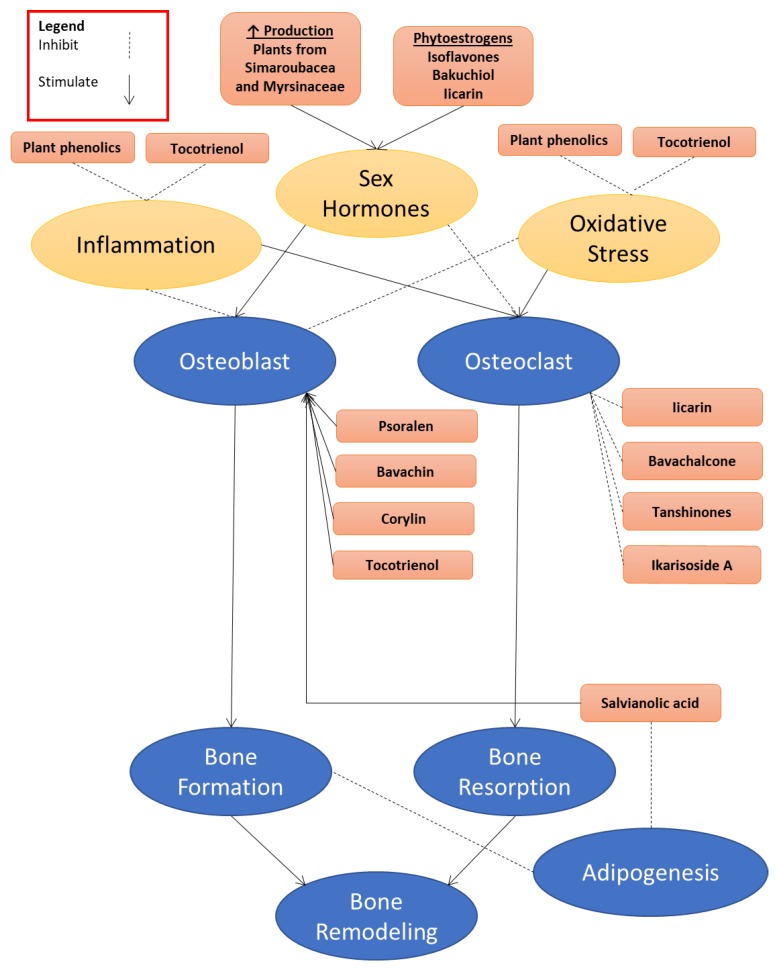
The role of botanical bioactive compounds in regulating bone metabolism. They may act directly on the bone cells, or through reducing inflammation and oxidative stress, or indirectly via increasing the level of sex hormones and interacting with sex hormone receptors on bone cells.

Herbal plants are a rich source of medicinal compounds that can be used to prevent osteoporosis. Many animal and cellular studies have been conducted to demonstrate the antiosteoporotic effects of these botanical extracts and their bioactive compounds (Table 1). They modulate bone remodelling by acting directly on the bone cells or through lowering oxidative stress and inflammation or increasing sex hormone levels (Figure 1). Through enhancing bone formation and suppressing bone reabsorption, these agents can improve bone mass and reduce the risk of fragility fracture. Fracture prevention also relies on improvements in muscle strength, coordination, and cognitive function. Botanical agents may affect these bodily functions, but they are outside the scope of this review. A proper human clinical trial to validate their bone-protective effects needs to be conducted. The use of botanical compounds as an intervention for osteoporosis also faces issues of standardization, selectivity, and safety. These issues should be overcome to promote their use in preventing osteoporosis.

## Figures and Tables

**Table 1:** Summary of anti-osteoporotic properties of medicinal plants.

Family	Scientific Name	Compound	Pharmacological study
*Berberidaceae*	*E. brevicornum* Maxim *E. sagittatum* Maxim *E. pubescens* Maxim *E. koreanum* Nakai *E. koreanum*		➢ Prevents osteoporosis without causing uterine hyperplasia in ovariectomized rats. ➢ Inhibits bone resorption, triggers bone formation, and blocks urinary calcium excretion. ➢ Increases the messenger ribonucleic acid expressions of bone morphogenetic protein and wingless-type signaling pathway related regulators such as bone morphogenetic protein-2 and cyclin D. ➢ Stimulates osteoblast proliferation via estrogen receptor-dependent mechanism. ➢ Possesses estrogenic activity and is able to regulate bone metabolism and improve the maturation of osteoblasts by inducing alkaline phosphatase, bone morphogenetic protein-2, macrophage colony stimulating factor, osteoprotegerin, receptor activator of nuclear factor-κB ligand, core binding factor α1, and interliukin-6 and signaling effectors against decapentaplegic protein 4.
		Iicarin	➢ Inhibits bone loss in the distal femur and tibia of the rat model and postmenopausal women. ➢ Decreases tartrate-resistant acid phosphatase activity of osteoclasts, decreases the size of lipopolysaccharide-induced osteoclasts formation, prevents lipopolysaccharide-induced bone resorption and interleukin-6 and tumor necrosis factor-α expression. ➢ Inhibits cyclooxygenasetype-2 synthesis, expression of lipopolysaccharide-induced hypoxia inducible factor-1α, and lipopolysaccharide-mediated activation of the p38 and Jun N-terminal kinase involved in osteoclasts differentiation. ➢ Reduces extracellular regulated-kinases 1/2 and lipopolysaccharide-induced activation. ➢ Reduces specific genes of osteoclasts: tartrate-resistant acid phosphatase, matrix metalloproteinase-9, cathepsin K and receptor activator of nuclear factor-kappa-B ligand.
		Ikarisoside A	➢ Shows antioxidant and anti-inflammatory properties in lipopolysaccharide-stimulated bone marrow-derived macrophage precursor cells and in RAW264.7 cells. ➢ Inhibits activation of nuclear factor kappa-light-chain-enhancer of activated B cells, Jun N-terminal kinase, protein kinase B-receptor activator of nuclear factor-κB ligand pathway in osteoclasts and their resorbing activity.
*Fabaceae*	*Glycine max* L. *Psoralea corylifolia* L.		➢ Dietary soybean protein supplementation is effective in reducing loss of bone mineral density in ovariectomized rats. ➢ Improves bone turnover markers, bone mineral density, and bone strength among postmenopausal women. ➢ Modulates bone metabolism-related gene expression of collagen type I, osteocalcin, calciotropic receptor, alkaline phosphatase, cytokines, and growth factors. ➢ Induces bone calcification in rats. ➢ Increases the concentration of inorganic phosphorus in serum. ➢ Regulates the trabecular microstructure and prevent bone loss in postmenopausal women and animal models.
		Genistein	➢ Shows estrogenic effects in the bone but not in the uterus. ➢ Modulates B-lymphopoiesis. ➢ Inhibits bone degradation.
		Bavachalcone	➢ Inhibits osteoclastogenesis. ➢ Inhibits the extracellular regulated-kinases and protein kinase B signalling and chromosome-Fos and nuclear factor of activated T cells c1 induction during differentiation.
		Psoralidin, Isobavachin	➢ Strong antioxidant.
		Bavachin Corylin	➢ Stimulates osteoblastic proliferation.
		Bakuchiol	➢ Has high binding affinity for ERα. ➢ Shows no significant uterotrophic activity. ➢ Stimulates estrogenic activity in vitro. ➢ Reduces postmenopausal bone loss by increasing alkaline phosphatase, calcium concentrations, serum estrogen concentration, and bone mineral density.
		Psoralen	➢ Stimulates new bone formation. ➢ Stimulates differentiation of osteoblasts in a dose-dependent manner in primary mouse calvariae. ➢ Upregulates osteoblast-specific genes expression of osteocalcin, type I collagen and sialoprotein. ➢ Stimulates bone morphogenetic protein-2 and bone morphogenetic protein-4 gene expression.
*Arecaceae*	*Elaeis guineensis*	Tocotrienol	➢ Well-known for their antioxidant, anti-oxidative stress, anti-inflammatory properties and anti-osteoporotic agent. ➢ Suppresses the proinflammatory cytokines expression. ➢ Effective in retaining trabecular bone structure in the nicotine-induced bone loss model. ➢ Reduces of single-labelled surface and increased in double-labelled surface in the ovariectomized rats. ➢ Increases bone mineral density at the femur and vertebrae of the rats in the testosterone deficiency and the glucocorticoid bone loss model. ➢ Restores bone calcium level at the femur and vertebra of orchidectomized and ovariectomized rats. ➢ Improves biomechanical strength of the femur in normal male rats.
*Labiatae*	*Salvia miltiorrhiza* Bunge		In ovariectomized rats: ➢ Prevents the decrease in trabecular bone mass and bone mineral density. ➢ Reduces the tartrate-resistant acid phosphatase activity. ➢ Decreases oxidative stress.
		Tanshinones	➢ Reduces the tartrate-resistant acid phosphatase-positive multinucleated osteoclast formation
		Tanshinones IIA	➢ Partially inhibits ovariectomy-induced bone loss by reducing bone turnover.
		Salvianolic acid A	➢ Inhibits bone loss in rats given long-term prednisone. ➢ Stimulates osteogenesis. ➢ Suppresses adipogenesis in bone marrow stromal cells.
		Salvianolic acid B	➢ Inhibits glucocorticoid-induced cancellous bone loss. ➢ Suppresses adipogenesis. ➢ Stimulates bone marrow stromal cell differentiation to osteoblasts. ➢ Upregulates osteoblastic activities. ➢ Modulates the expression of messenger of ribonucleic acid of dickkopf-1, runt-related transcription factor 2, peroxisome proliferator-activated receptor gamma, and β-catenin in mesenchymal stem cell.
*Simaroubaceaea*	*Eurycoma longifolia*		➢ Androgenic substance with a good safety profile.
		Eurycomalactone Eurycomanol	➢ Increases testosterone level in the blood. ➢ Inhibits sex hormone-binding globulin.
		Eurycomanone	➢ Increases testosterone level in the blood.
*Myrsinaceae*	*Labisia pumila*		➢ Used traditionally to treat female sexual problems. ➢ Stimulates the production of estrogen. ➢ Stimulates the production of estrogen.
		Ascorbic acid Anthocyanin Beta-carotene, Flavonoids phenolic compounds	➢ Anti-oxidant and free radical scavengers-effective free radical scavengers in conditions, such as osteoporosis and rheumatism, which are related to ageing and oxidative stress. ➢ Anti-inflammatory agents.

## Abbreviations

ALP	Alkaline phosphatase
BMP-2/4	Bone morphogenetic protein-2/4
M-CSF	Macrophage colony stimulating factor
OPG	Osteoprotegerin
RANKL	Receptor activator of nuclear factor-κB ligand
Cbfα1	Core binding factorα1
SMAD4	Signaling effectors mothers against decapentaplegic protein 4
Wnt-signaling	Wingless-type signaling
cyclinD	Cyclin dependent
OVX	Ovariectomized
ER	Estrogen receptor
TRAP	Tartrate-resistant acid phosphatase
LPS	Lipopolysaccharides
IL-6/1	Interleukin-6/1
TNF-α	Tumor necrosis factor
COX-2	Cyclooxygenasetype-2
HIF-1α	Hypoxia inducible factor-1*α*
p38	Protein 38
JNK	Jun N-terminal kinase
ERK1/2	Extracellular regulated-kinases 1/2
Iκ-BαLPS	ikappa-Balpha lipopolysaccharide
MMP-9	Matrix metalloproteinase-9
Akt	Protein Kinase B
NF-κB	nuclear factor kappa-light-chain-enhancer of activated B cells
RANK	receptor activator of NF-κB
COL I	collagen type I
NFATc1	nuclear factor of activated T cells c1
c-Fos	Chromosome-Fos
B-lymphopoiesis	Bone marrow-lymphopoiesis
ERα/ERβ	Estrogen receptor alpha/beta
BMD	Bone mineral density
Osx	Osteoblast-specific transcription factor osterix
MDA	malondialdehyde
NO	nitric oxide
mRNA	Messenger ribonucleic acid
Dickkopf-1	DKK-1
Runx2	Runt-related transcription factor 2
PPAR-γ	Peroxisome proliferator-activated receptor gamma
β-catenin	Beta-cateni
MSC	Mesenchymal stem cell
EL	*Eurycoma longifolia*
ERT	estrogen replacement therapy
